# The Significance of Tau Aggregates in the Human Brain

**DOI:** 10.3390/brainsci10120972

**Published:** 2020-12-11

**Authors:** Rudy J. Castellani

**Affiliations:** 1 Department of Pathology, Anatomy, and Laboratory Medicine, West Virginia University, Morgantown, WV 26506, USA; rudolph.castellani@hsc.wvu.edu; 2 Department of Neuroscience, Rockefeller Neuroscience Institute, West Virginia University, Morgantown, WV 26506, USA

**Keywords:** tau, tauopathy, neurofibrillary, Alzheimer’s disease

## Abstract

Neurofibrillary degeneration has attracted the attention of neuroscientists as both a hallmark of the disease and a subject for experimentation for more than a century. Recent studies implicate phosphorylated tau (p-tau) directly in neurodegenerative disease pathogenesis, although the human data continue to raise questions. P-tau accumulates with age in a roughly hierarchical manner, but avoids abundance in the neocortex unless co-occurring with amyloid-β. Neurodegenerative tauopathies tend to have p-tau morphologies that differ from aging and Alzheimer’s disease. Tau isoforms (3R vs. 4R) have a tendency to vary with tauopathy phenotype for unknown reasons. Selective vulnerability to p-tau and spatial-temporal disconnect from amyloid-β are evident in aging. P-tau assessment at autopsy involves tissue decomposition, which may skew microanatomical observations toward limited biological meaning. Two major consensus guidelines for interpreting p-tau at autopsy emphasize the challenges of clinicopathologic correlation, and reinforce the observation that regional neurodegeneration is a better correlate of clinical signs than is proteinopathy. Despite the proliferation of interesting and novel theories related to tau-mediated pathogenesis, the weight of the human observations suggests that neurofibrillary degeneration is an epiphenomenal hallmark of aging and disease rather than an epicenter of neurotoxicity. This is consistent with numerous tau-targeted therapeutic strategies that have been unsuccessful to date.

## 1. Introduction

Neurofibrillary degeneration was first identified by Alzheimer in his seminal case report linking microscopic lesions to progressive neurological decline [1]. In doing so, he established Alzheimer’s disease (AD) as an entity and neurodegeneration as a category of disease. Much has been learned about AD since then, although the lesions in question remain the same. Indeed, the silver technique used by Alzheimer is still used today to identify the same hallmark lesions [2,3].

Researchers in the early and mid-20th century described neurofibrillary degeneration in copious detail, but were circumspect about its significance with respect to the disease process. Bielschowsky commented that alterations in nerve cells indicate only that “pathological processes have taken place” [4]. Malamud in 1929 concluded from his case series that “etiologically, this clinicopathologic syndrome may be caused by a variety of factors,” and that “the whole process has been so thoroughly completed that a possible pathogenetic theory of it could only be guessed at” [5]. McMenemey in 1940 suggested the changes “as a permanent tombstone to mark the site of the deceased cell” [6]. King noted in 1942 that “the nature and origin of this pathological material remain unsettled” [7]. Early reports on electron microscopy of neurofibrillary change by Kidd [8] and Terry [9,10] were purely descriptive in nature, with Kidd commenting that “it is difficult to speculate on the nature of these structures.” Hirano did not attach special significance to neurofibrillary tangles but instead listed populations of neurons that appeared vulnerable [11].

The paradigm shifted in the mid-1980s as researchers identified individual proteins within microscopic lesions. Brion et al. [12] first reported tau protein (named for its role in polymerization of tubulin [13]), as a major protein component of neurofibrillary tangles. Grundke-Iqbal et al. [14] reported similar findings shortly thereafter and commented that post-translational modifications such as phosphorylation may be driving the histogenesis of neurofibrillary change. Kinase-phosphatase biology would soon follow [15]. Both phosphatase modifiers and kinase inhibitors are in clinical trials today for Alzheimer’s disease (AD) therapeutics [16]. In essence, the hallmark lesion itself has been conceptualized in recent years as an epicenter of pathogenesis and a potential target for therapy.

The purpose of this review is to explore the subject of p-tau lesions as they present in the human brain, in both aging and neurodegenerative diseases. The spectrum of p-tau lesions, which has expanded considerably in recent years, has added a level of complexity, not only morphologically, but in terms of a putative role in various disease states. The central question at issue is the nature and significance of p-tau aggregates identified at autopsy, and whether the lesions themselves and their constituent proteins drive neurodegenerative diseases.

## 2. Phosphorylated Tau Assessment In Situ

The term “tauopathy” was first used for a condition with a mutation in the tau (*MAPT*) gene [17], which seems appropriate since tau protein itself is mutated in this condition and associated with autosomal dominant disease. Over time, the term “tauopathy” generalized to refer to the simple presence of p-tau aggregates identified by immunohistochemistry, whether or not the aggregates occurred in the context of clinical disease.

Because p-tau occurs with age starting as early as childhood, it is important to distinguish incidental or subclinical tauopathy, i.e., tauopathy with no clear clinical correlate, from neurodegenerative tauopathy, i.e., a well-defined clinicopathologic entity with progressive neurodegeneration. (Table 1). Immunohistochemical phenomena such as argyrophilic grain disease [18], aging-related tau astrogliopathy [19], primary age-related tauopathy [20], and chronic traumatic encephalopathy (2005 forward [21,22]), are examples of subclinical tauopathy insofar as they lack a consistent clinical phenotype and are diagnosed exclusively post-mortem. Progressive supranuclear palsy, corticobasal degeneration, and Pick disease are neurodegenerative tauopathies, as they invariably correlate with clinically progressive neurodegenerative disease [23]. These are sometimes referred to as “primary tauopathies” (as opposed to a “secondary tauopathy” such as Alzheimer’s disease), although it remains unclear as to whether p-tau is driving the disease process in these conditions, occurs in parallel with neurodegeneration, or is otherwise secondary to more complex biology. Exceedingly rare tauopathies with complex nosology and variable clinical presentations also appear to be neurodegenerative in nature (e.g., globular glial tauopathy, atypical Parkinsonism of Guadeloupe, diffuse neurofilament tangles with calcification [24]).

P-tau aggregates are not only age-related, but an inevitable consequence of the aging process [25,26]. They are sparse in children and young adults, but nevertheless detectable. P-tau can be strikingly abundant in middle-aged and older people in the absence of a neurocognitive disorder during life [27]. Thus, in a random autopsy sample across the spectrum of ages, p-tau aggregates are unassociated with clinical disease in the overwhelming majority of cases, with most of the rest being AD-related.

Numerous different p-tau antibodies are available for brain tissue examination in situ, which reflects the large number of tau phosphorylation sites and candidate p-tau epitopes [28,29]. This is a potentially important issue because the antigenicity of different epitopes varies as a function of time and cell viability. Some antibodies better label “pretangles” (more diffuse cytoplasmic labeling with no apparent fibrils), while others may be more effective at labeling extracellular tangles (neurofibrillary tangles that persist in tissues following cell death) [30]. In practice, monoclonal antibody AT8 is commonly used because it recognizes residues in both intracellular and extracellular lesions, and does not cross react with unphosphorylated tau [30], providing robust sensitivity and a clean background.

**Figure 1:**
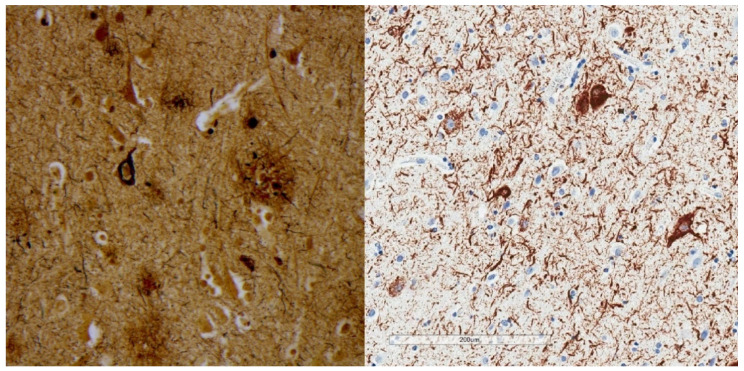
Bielschowsky silver stain (**left**) shows argyrophilia involving a neurofibrillary tangle and a neuritic plaque. P-tau immunohistochemistry using monoclonal antibody AT8 (**right**) shows neurofibrillary tangles and numerous neuropil threads.

P-tau aggregates present a broad array of descriptive morphologies or intracellular “inclusions” that have expanded well beyond neurofibrillary degeneration (Table 2) (reviewed in [31,32]). Prior to immunohistochemistry, silver reduction reactions were generally used (Figure 1). The latter requires an underlying protein complex with filamentous or fibrillar structure, and were adapted empirically for the structures of interest. For example, the Bielshowsky technique which employs silver nitrate is more sensitive for neurofibrillary tangles than axonal cytoskeleton [33], whereas the reverse is true for the Bodian method [34], which employs silver proteinate and copper. Immunohistochemistry adds the ability to probe for specific proteins and specific epitopes on those proteins and is inherently more sensitive and specific for tau. A subset of p-tau morphologic variations are minimally apparent or not apparent at all with silver impregnation [21].

**Figure 2:**
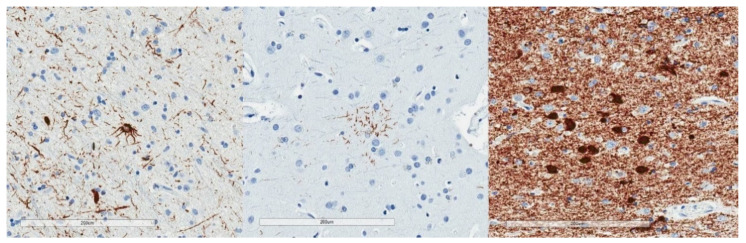
P-tau (AT8) immunohistochemical stains demonstrate a tufted astrocyte (**left**), an astrocytic plaque (middle), and Pick bodies (**right**). These morphologies are reasonably specific for progressive supranuclear palsy, corticobasal degeneration, and Pick disease, respectively.

Neurodegenerative tauopathies tend to have p-tau lesions with implied specificity, which is less the case with subclinical tauopathies. Astrocytic plaques and tufted astrocytes, for example, are relatively specific for corticobasal degeneration and progressive supranuclear palsy respectively [35]. Pick bodies are limited to Pick disease for practical purposes (Figure 2). In contrast, the subclinical tauopathies accumulate p-tau morphologic aggregates that occur with age (neurofibrillary tangles, pretangles, thorn-shaped astrocytes, threads, grains), differing only in a regional pattern or relative abundance.

Interobserver variability and diagnostic accuracy of the various inclusions have generally been unexplored, although the diagnostic process in neurodegenerative tauopathies is not confined to p-tau inclusions. Diagnosis takes into account clinical presentation and regional neurodegeneration. Proteinopathy per se correlates poorly with neurological deficits in neurodegenerative diseases compared to regional neurodegeneration or atrophy [23,36], notwithstanding complex distribution patterns and proposed staging in some tauopathies [37].

Human tau protein has been studied in some detail with respect to alternative splicing, most notably exon 10, which generates three or four ~32 amino acid repeats (3R and 4R tau) in the microtubule-binding domain. Detailed discussions are available elsewhere [31]. Alternative splicing may be reflected in the relative predominance of tau isoforms in the various p-tau lesions (Table 3). For example, lesions in progressive supranuclear palsy and corticobasal degeneration have relatively more 4R tau. Pick disease has relatively more 3R tau. Subclinical tauopathies may be either 4R or 3R + 4R tauopathies.

## 3. Tau in Aging and Alzheimer’s Disease

**Figure 3:**
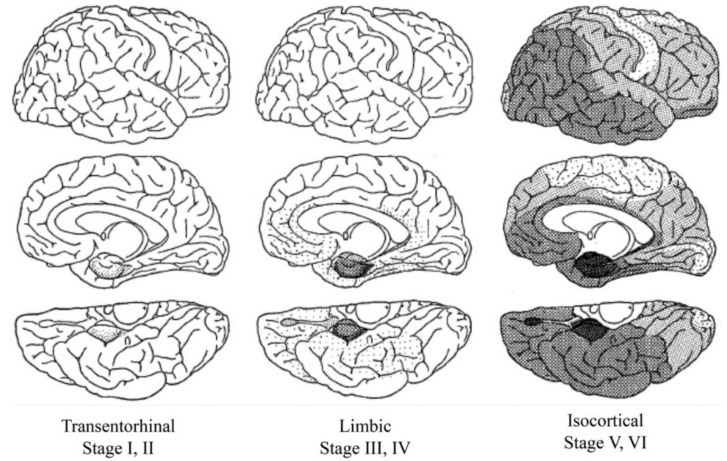
Schematic representation of Braak stages [39]. Note that p-tau in the isocortical stages is usually accompanied by amyloid-β deposits and neurocognitive dysfunction. Braak stages I through IV in the absence of amyloid-β has no clear association with clinical signs. Reproduced with permission from Springer.

P-tau accumulation tends to occur in a stereotyped fashion [38,39], conceptualized as so-called Braak stages (Figure 3). P-tau appears first in the locus ceruleus, followed by the transentorhinal region (e.g., stage I and II), limbic pathways (e.g., stage III and IV), and isocortex (Braak stages V and VI). Primary motor and sensory neocortical areas are relatively spared in the Braak scheme, even in advanced AD [38,39]. P-tau in subcortical areas other than the locus ceruleus occurs with age, but is not otherwise adopted for staging purposes.

Involvement of the locus ceruleus is interesting in that (i) immunolabeling for p-tau appears as early as childhood [26] and (ii) the locus ceruleus has more diverse neuroanatomical connections than any other brain region [40]. These basic observations tend to contradict prion-like p-tau templating and neuroanatomical spread as a meaningful neurodegenerative process *in vivo*, at least as regards human aging.

**Figure 4:**
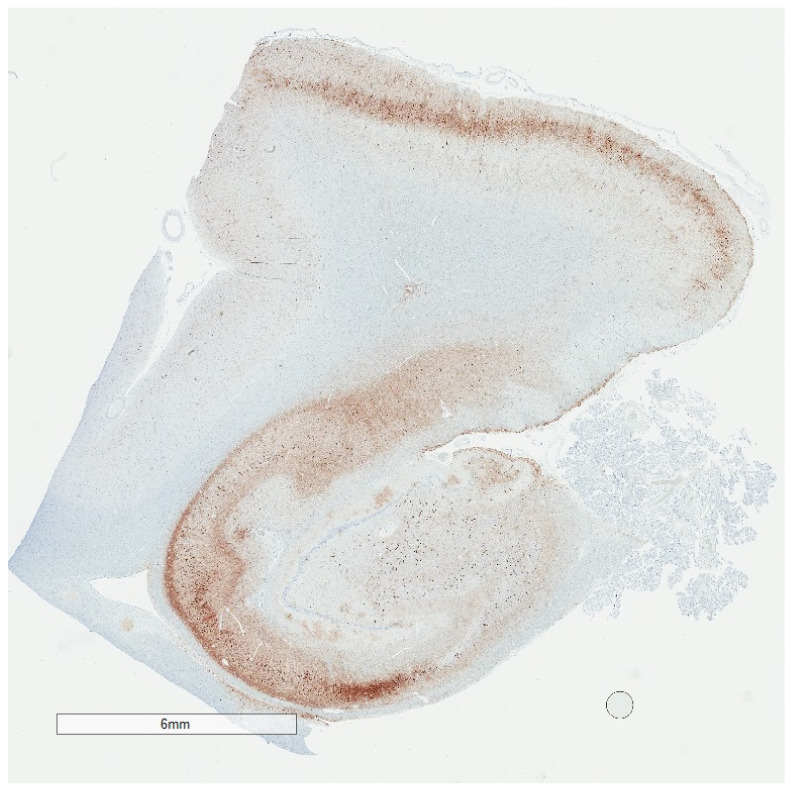
Low magnification p-tau (AT8) immunohistochemistry of the hippocampus showing changes consistent with Braak stage IV. Despite the extensive p-tau labeling, individuals with Braak stage IV may have been cognitively normal during life.

While the Braak scheme tends to suggest that p-tau progresses in all people from the brainstem to medial temporal lobe to neocortical areas given enough time, in reality, Braak stages are a composite of healthy aging and AD. Braak stages I through IV in the absence of Aβ are both common and strictly age-related [20,41]. Braak stage III and IV without Aβ (Figure 4), may be encountered in cognitively intact centenarians, for example [42]. Braak stage V/VI is for practical purposes *de facto* evidence of co-existing Aβ and some neurocognitive deficit [43,44]. There are no data as yet to suggest that people with medial temporal p-tau aggregates without Aβ will invariably progress to full-blown dementia, although once initiated, AD encompasses medial temporal p-tau aggregates as well as Aβ pathology [2,3]. In this sense, p-tau progression through Braak stage IV in the absence of Aβ seems to distinguish “senility” from AD.

P-tau is generally viewed as more closely aligned with clinical signs than is Aβ. Precise clinicopathologic data in support of this view are lacking, although general observations may be cited [45]. One observation is that Braak stages I-IV selectively involve memory circuitry (entorhinal/transentorhinal region and Ammon’s horn) [38,39]. Since memory dysfunction is among the early clinical manifestations of AD, p-tau in these locations may be viewed as evidence of clinical correlation. A problem with this concept is that p-tau in memory circuitry is common in control brains, in some cases abundant, with no precise association with clinical signs during life [20]. 

A second observation is that Aβ plaques are occasionally abundant in cognitively intact elderly [43,46], while an abundance of neocortical p-tau is reasonably predictive of cognitive dysfunction [45]. Aβ thus appears to be a more sensitive indicator of the process of AD, whereas p-tau appears to be more specific, but only when present in the neocortex and in considerable abundance. Qualitatively, neither p-tau nor Aβ is specific for clinical signs. This does not preclude the possibility that certain p-tau species may show more or less viability as a disease biomarker once developed. For example, p-tau217 in a recent study appeared to correlate better with other measures of Alzheimer’s disease burden than p-tau181 [47]. In terms of pathogenic significance, such correlations are only possible in advanced disease, when neuronal loss by whatever mechanism may be just as meaningful [46].

## 4. Selective Vulnerability

P-tau accumulation consistently involves certain neuronal populations in normal aging, such as the locus ceruleus, the amygdala, and the entorhinal cortex, while nearby neuronal populations may be uninvolved. Hirano observed nearly 60 years ago that mesencephalic neuronal populations were in general severely affected in the Dementia–Parkinson complex of Guam, yet the mesencephalic nucleus of the trigeminal nerve was completely spared [11]. The cerebellar cortex does not contain p-tau aggregates at all, for practical purposes, regardless of advanced age or AD [48]. The biological basis for such “selective vulnerability” is unclear.

**Figure 5:**
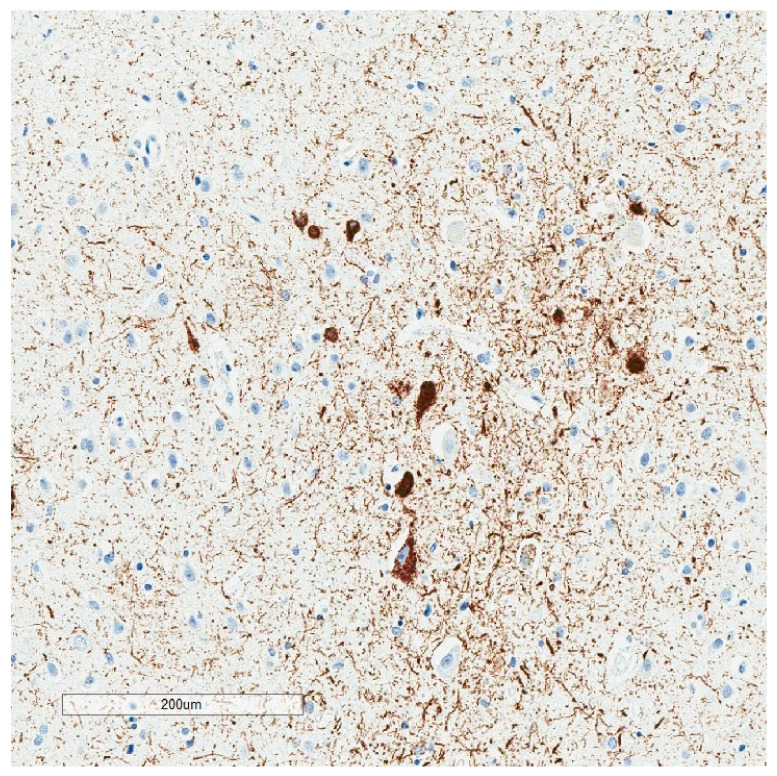
Selective labeling of the pre-alpha neurons of the entorhinal cortex by p-tau (AT8) immunohistochemistry, highlighting selective vulnerability.

Selectivity is plainly apparent in the aging process, in which deposits may be limited to the locus ceruleus or the pre-alpha neurons in the entorhinal cortex [26] (Figure 5), for example. P-tau in these early sites also occurs independent of Aβ deposits. When Aβ deposits do occur, they typically appear later in life and first appear in the neocortex [26], followed by allocortex, subcortical structures, and cerebellum. It is interesting in this respect that Aβ deposits in autopsy brains proceed from neocortical to allocortical to subcortical, which is the opposite of p-tau progression. Some have suggested that this runs counter to the amyloid cascade theory, which proposes Aβ as the initiator and p-tau as a consequence, in the same microenvironment [49,50].

## 5. To What Extent Is p-Tau Neuropathology an Artifact of Decomposition?

The experience and expertise of neuropathologists with respect to p-tau and the human brain are derived almost entirely from autopsy brain examination. Because autolysis begins immediately after death, a level of decomposition is axiomatic. Autolysis involves enzyme activity, including phosphatases, and indeed p-tau rapidly dephosphorylates post-mortem [51,52]. P-tau antibodies therefore preferentially label buried epitopes. In addition, chemical denaturation (antigen retrieval) is routinely employed so that antibody probes can access those buried p-tau epitopes. P-tau aggregates identified post-mortem are also protease insoluble [53]. It seems plausible in this context that p-tau lesions identified at autopsy are relatively inert.

The insolubility of p-tau aggregates detected post-mortem may have implications with respect to PET scanning. The ability of tau PET tracers to access p-tau aggregates in a manner comparable to post-mortem immunolabeling has been shown anecdotally [54], although a precise correlation between tau PET signals in living organisms and p-tau neuropathology seems doubtful. Existing tau tracers are not specific to tau itself but instead beta-sheet conformation [55], common to a number of proteins. In addition, chemical denaturants such as formic acid specifically disrupt tau tracer binding [56], while formic acid and other denaturants actually enhance p-tau antibody binding. Target ligands for tau PET tracers and p-tau antibodies may therefore be mutually exclusive at the molecular level.

The issue of p-tau neuropathology as insoluble post-mortem aggregates is also relevant to the two areas of neurotoxicity theory that have predominated in recent years—oligomer biology [57] and prion-like protein templating [58]. The depth and breadth of these areas of inquiry are beyond the scope of this review, but mentioned here only insofar as both subjects emphasize small molecules as biomarkers, rather than insoluble inclusions. The toxicity of tau oligomers, for example, is linked to their solubility [53]. Hallmark lesions are epiphenomenal by definition in these constructs, perhaps formed en passant while the primary disease process is taking place, or at most a surrogate for pathogenic processes indiscernible to the light microscope.

## 6. Are p-Tau Aggregates in Part Evanescent?

**Figure 6:**
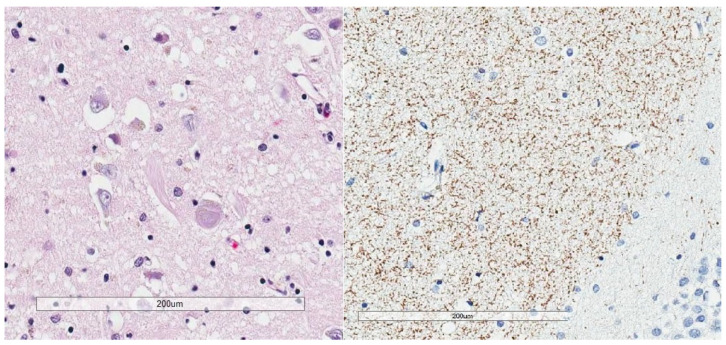
The kinetics of p-tau labeling over time is generally unclear because autopsy examination is a single snapshot in time. Extracellular or “ghost” neurofibrillary tangles (**left**) are evidence that neurofibrillary tangles are resistant to degradation, with some studies suggesting that they persist in brain tissue for decades. On the other hand, dendritic labeling by p-tau immunohistochemistry in the stratum lacunosum moleculare (**right**) may be transient.

NFTs in AD and aging consist of densely fibrillar material by light microscopy, and paired helical filaments ultrastructurally. They are protease insoluble as noted, so it makes sense that NFT’s persist in cells for decades [59]. Extracellular NFTs remain detectable in neuropil after cell death, and retain argyrophilia as well as variable immunoreactivity to p-tau [30] (Figure 6).

**Figure 7:**
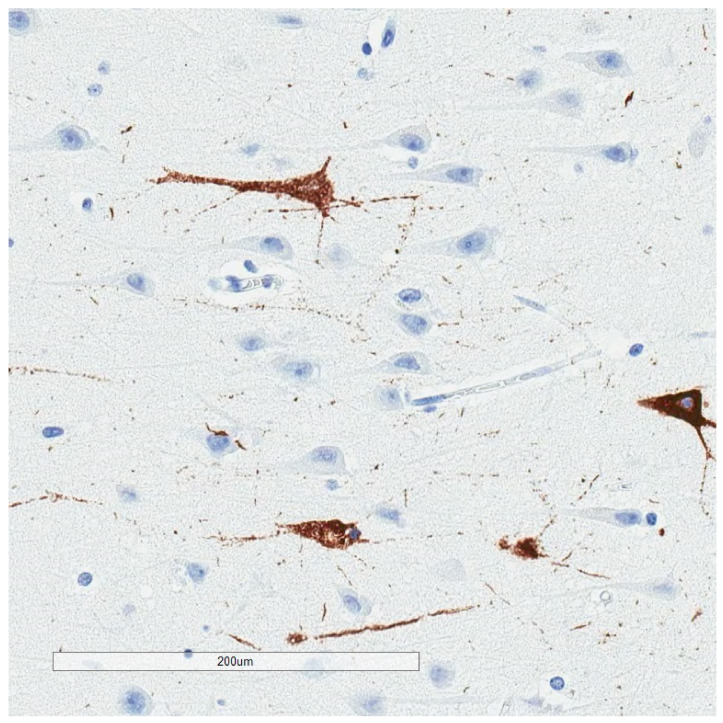
Neuronal pretangles show more diffuse p-tau labeling in the neuronal perikarya. Pretangles tend to lack fibrillarity and argyrophilia.

Since the availability of p-tau immunohistochemistry, the spectrum of lesions has expanded to include the “pretangle” noted above. Pretangles are non-fibrillar, non-argyrophilic expressions of p-tau within neuronal perikarya (Figure 7). One presumption would be that pretangles represent an early stage in neurofibrillary degeneration. An interesting related question would also be the extent to which p-tau is resorbed through endogenous protease digestion or is otherwise transient. For example, transient pretangle-like p-tau involving dendrites of the stratum lacunosum moleculare and CA-1 region is well described [38,60] (Figure 6). It is plausible that sparse non-argyrophilic p-tau such as described in some people with repetitive head impact exposure [21] is in part evanescent, similar to dendritic p-tau in the hippocampus.

## 7. P-Tau Lesions as Targets for Translational Research

The focus on NFTs in translational research is due primarily to their densely fibrillar structure, which in turn reflects extensive protein aggregation to the point of being visible by light microscopy. The latter quality, the simple fact that NFTs can be visualized by light microscopy, was the limiting factor for the identification of tau within NFT and therefore the emergence of tau as a primary pathogenic theory. If tau pathophysiology were strictly a soluble phase or functional phenomenon and otherwise invisible to morphological analysis, attention would have been directed elsewhere. The first question to ask, therefore, when advancing hypotheses based on in situ observations of NFT, is whether tau metabolism is under study versus some epiphenomenon of massive protein aggregation. For example, NFTs have been shown to co-label redox-active iron [61], multiple protein adducts associated with oxidative stress [62,63,64], proteins that drive the cell cycle [65], heme-oxygenase [66], mammalian target of rapamycin [67], and markers of caspase cleavage [68], among other molecules. Post-translational modifications such as O-glycosylation, advanced glycation, ubiquitination, nitration, SUMOylation, prolyl-isomerization, and truncation have also been observed in situ [69,70,71,72,73,74,75]. Whether some or all of these observations are related to tau metabolism or some other aspect of the macromolecular complex is an open question. 

Other in situ changes co-occur with NFTs without co-labeling. For example, 8-hydroxyguanosine, an oxidative stress-induced nucleic acid modification, specifically avoided NFT-bearing neurons in one study [76]. Possible induction of apoptosis via mitochondrial dysfunction, calcium influx, and caspase activation is described, as occurring in the Alzheimer’s disease microenvironment but not necessarily co-localizing with NFTs [77]. Another study raised the question of whether neuronal loss proceeds through stress granule biology without ever reaching the NFT stage [78]. From a functional standpoint, one study demonstrated NFT accumulation in a mouse model while memory deficits were rescued [79], as evidence of a potential disconnect between NFT accumulation and clinical function.

Taken together, in situ studies suggest that NFTs have their own complex biology that extends beyond tau structure, splice variants, and phosphorylation state, and that the biology of tauopathies extends well beyond NFT, with myriad potential driving factors and the possibility of some neuronal loss in non-NFT pathways. NFTs and associated p-tau have thus served as the nidus for broad pathogenic theory, which over time has tended to substantiate the notion of p-tau aggregates as a marker of disease, and deplete the notion that p-tau is etiological. This is consistent with attempts at intervention with tau-targeted strategies or lesion-targeted strategies in general [80], which have proven to be challenging [81].

## 8. Consensus Guidelines for p-Tau Research: Alzheimer’s Disease versus Chronic Traumatic Encephalopathy

The most recent AD criteria (National Institute on Aging—Alzheimer Association, or NIA-AA criteria) employ Braak staging for p-tau, and Aβ assessment using Thal amyloid phases and CERAD neuritic plaque scores [2,3]. The output of the criteria is an “ABC” score—Amyloid phase, Braak stage, and CERAD plaque score—of “Alzheimer’s disease neuropathologic change,” each component semi-quantitated as 0 to 3. An interpretation of “Alzheimer’s disease neuropathologic change A1, B1, C1” would be an example of mild pathology, while an interpretation of “Alzheimer’s disease neuropathologic change A3, B3, C3” would be an example of advanced pathology.

The NIA-AA criteria are not used to predict neurological signs in the absence of clinical data. In fact, the consensus group commented specifically that the updated criteria are intended to “disentangle” Alzheimer’s disease neuropathologic change from the neurological assessment, given the long known challenges in clinicopathological correlation [2]. The consensus article does provide guidelines for establishing AD as an entity, but only for cases with known dementia during life. Thus, given the presence of dementia, an intermediate degree or more of *both* p-tau pathology and Aβ pathology is sufficient to conclude that a decedent’s clinical dementia can be assigned to the clinicopathologic entity of Alzheimer’s disease. Embedded in these criteria is that p-tau pathology is by itself insufficient to explain AD dementia without at least intermediate Aβ pathology, which reinforces the concept that p-tau alone, even in abundance, may be devoid of a predictable clinical correlation.

The NINDS/NIBIB consensus effort for chronic traumatic encephalopathy is the only such effort to date that is centered on p-tau predominantly. The criteria were based on a study of ten presumed CTE cases (all contact sport athletes) and fifteen non-CTE tauopathy cases [21], interpreted by consensus invitees with expertise in neurodegenerative diseases. The group was given *a priori* criteria for CTE which were modified over the course of the study. The group was aware of the finite list of presumptive diagnoses, but was otherwise blinded to clinical information. The study reported a good agreement (Cohen’s kappa 0.78) on the diagnosis of CTE, although there were cases in which some experts diagnosed CTE in non-CTE cases and vice versa. The study did not address whether specific neuropathology correlated with specific neurological signs, and did not adopt CTE stages.

**Figure 8:**
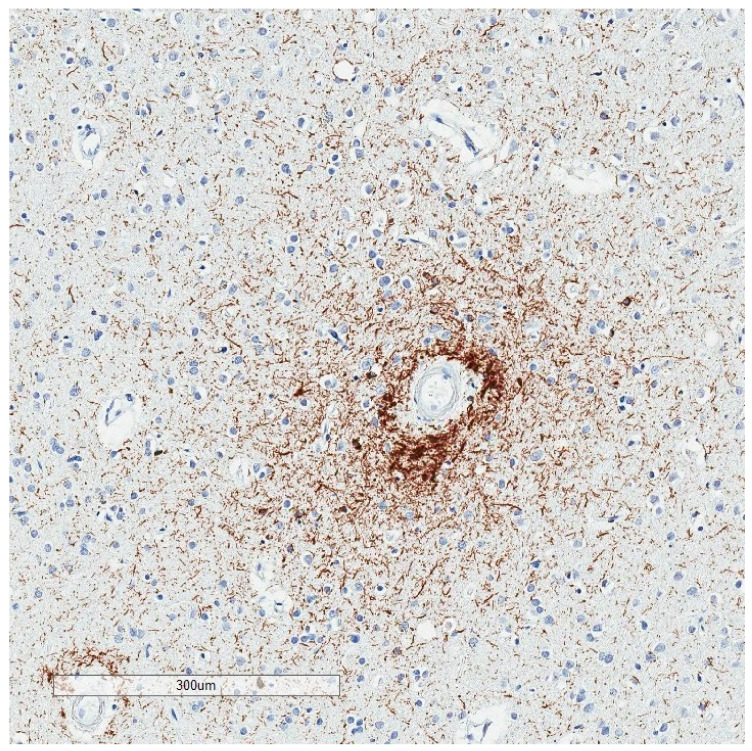
Irregular p-tau aggregates with “vasculocentric neurites” at a sulcal depth, consistent with the required criterion for CTE. The decedent was a 78 year old man with Parkinson’s disease and Alzheimer’s disease pathology. His traumatic brain injury and contact sport history are unknown.

There is broad overlap in tissue sampling between the NINDS/NIBIB consensus and the NIA-AA guidelines, but there are otherwise a number of fundamental differences between the two sets of guidelines (Table 4): (i) The NINDS/NIBIB paper did not define an upper limit to the extent of sampling and p-tau immunostaining. A minimum of 11 brain regions was recommended, and three additional regions if high suspicion, with the caveat that 20% of cases might be missed by this approach, without, for example, sledge microtome-obtained immunohistochemical stains. In theory one could subject the entire brain to p-tau immunostains looking for the lesion of interest; (ii) The NINDS/NIBIB criteria propose a “pathognomonic lesion” (p-tau aggregates in neurons, astrocytes, and cell processes in an irregular distribution around small blood vessels at the depths of cortical sulci (Figure 8)), i.e., a diagnosis beyond doubt, that infers mechanism, clinical disease, and a neurodegenerative process. The NIA-AA effort, in contrast, specifically avoided clinical inferences and frankly admitted that the disease mechanism is unknown [2]; (iii) Selective vulnerability to p-tau is proposed as a function of the contour of the cortical ribbon (sulcal depth) and vicinity to small blood vessels (“around small blood vessels”), rather than hierarchical involvement of neuronal populations; (iv) the required criterion lacks a lower threshold. One microscopic lesion is sufficient, irrespective of the clinical context. The NIA-AA guidelines require an abundance of two lesions in a specific context (dementia), as noted; (v) The NINDS/NIBIB criteria conceptualize neurodegenerative disease neuropathology and aging-related p-tau as co-morbidities. This appears to be of necessity because of the ubiquity of p-tau with age and the high frequency of neurodegenerative diseases among donors to brain banks. In contrast, NIA-AA guidelines incorporate only Lewy body diseases as co-morbidities. Other explanations for dementia, such as frontal temporal lobar degeneration, would indicate a different disease process and a different diagnosis.

The NINDS/NIBIB methodology and criteria appear useful as a screen for the lesion of interest, which will benefit the understanding of the pattern or patterns of immunoreactivity. Some caution might be warranted for clinical diagnostic interpretation, however. Each major neurodegenerative disease category includes subtypes with pathogenic mutations, as well as genetic polymorphisms that confer disease susceptibility. Interpreting neurodegenerative diseases as co-morbid to a CTE diagnosis runs the risk of assigning a genetic disorder to an environmental cause.

The appropriateness of either set of criteria as stand-alone guidelines for patient care is questionable. This was recognized in the 1991 CERAD article, which explicitly stated that the “protocol is not intended to characterize each case definitively” [82]. Likewise, the NINDS/NIBIB article points out that the proposed criteria are “preliminary” and a “first step” in the validation process [21]. The primary purpose of these consensus articles is more to facilitate research across institutions than to establish standards of practice for clinical diagnosis.

## 9. Conclusions

Neurofibrillary degeneration has attracted the attention of neuroscientists for many years and continues to do so. Its prominence historically had less to do with its pathogenic significance than the simple fact that it can be visualized with a light microscope, and as such can be correlated, characterized, and explored through experimentation. Neuropathologists have historically expressed reservations about neurofibrillary changes as an upstream event, with most favoring a reactive phenomenon in broader cellular pathobiology. Since the linkage of neurofibrillary changes to tau protein, researchers have been more accepting of primary neurotoxicity, which has manifested in pathogenic theories that tend toward functional biology and away from the structural lesions such as neurofibrillary tangles.

Some features of p-tau aggregates are noteworthy and may shed light on pathogenesis: (i) p-tau in isolation and in the absence of clinical or pathological neurodegeneration has no clinical correlates in the literature to date; (ii) p-tau even in abundance is insufficient as an explanation for AD dementia in the absence of other pathologies; (iii) selective vulnerability of neuronal populations to p-tau accumulation in the human brain is both striking and poorly understood. The most vulnerable population is also the most widespread in its functional connectivity, indicating benignity to p-tau in the aging process; (iv) the overlay of postmortem autolysis or the process of decomposition in assessing p-tau pathology may not be fully accounted for; (v) A subset of hippocampal p-tau immunoreactivity is demonstrably transient. This feature, and the frequency with which p-tau aggregates may be seen randomly in children and young adults, suggests that some p-tau lesions may be metabolized over time; (vi) consensus methodology and criteria for p-tau assessment at autopsy highlight the challenges of clinicopathologic correlation and are designed primarily to facilitate research across institutions.

The biological process of neurodegeneration remains a mystery. The various p-tau aggregates offer some insight into processes that have taken place, but a careful appraisal of p-tau neuropathology in the human brain does not permit the conclusion as yet that p-tau lesions drive neurodegenerative disease. More research is needed.

## Figures and Tables

**Table 1:** Examples of neurodegenerative tauopathy and subclinical tauopathy.

Neurodegenerative Tauopathies	Subclinical Tauopathies
Progressive supranuclear palsy	Primary age-related tauopathy
Corticobasal degeneration	Aging-related tau astrogliopathy
Pick disease	Argyrophilic grain disease
Chromosome-17-linked dementia	Chronic traumatic encephalopathy
Guamanian dementia	

Neurodegenerative tauopathies represent neurodegenerative diseases in the true sense, as invariably progressive conditions with neurological signs and abundant neuropathology that includes loss of neurons. Subclinical tauopathies lack clinical features of neurodegenerative disease and are strictly autopsy diagnoses.

**Table 2:** Morphologic variations of p-tau.

Neurofibrillary Tangle
Ghost (extracellular) tangle
Pretangle
Dystrophic neurite
Neuropil thread
Grain
Pick body
Tufted astrocyte
Equivocal tufted astrocyte
Coiled body
Astrocytic plaque
Globular glial inclusion
Ramified astrocyte
Thorny astrocyte
Bushy astrocyte
Fuzzy astrocyte

There are numerous and expanding microscopic lesions that label with antibodies to p-tau, most of which are not disease-specific. A subset is relatively specific (e.g., tufted astrocytes—progressive supranuclear palsy, astrocytic plaques—corticobasal degeneration, Pick body—Pick disease), although interobserver variability has not been explored, and neurodegeneration per se (loss of neurons, atrophy) is a better correlate of clinical signs.

**Table 3:** 3R versus 4R tauopathies.

Tauopathy	Isoform
Pick disease	3R
Parkinson-Dementia complex of Guam	3R+4R
Progressive supranuclear palsy	4R
Corticobasal degeneration	4R
Frontotemporal dementia & Parkinsonism linked to chromosome 17	Mixed
Primary age-related tauopathy	3R+4R
Chronic traumatic encephalopathy	3R+4R
Aging-related tau astrogliopathy	4R
Argyrophilic grain disease	4R

Tau protein may have three or four microtubule binding repeats (3R, 4R) due to alternative splicing of exon 10 of *MAPT*. 3R versus 4R tau may predominate depending on tauopathy phenotype, or they may be mixed (Arendt et al., 2016). Whether or how these differences relate to disease pathogenesis is unknown.

**Table 4:** Contrasts between NIA-AA 2012 (AD) and NINDS/NIBIB 2016 (CTE) guidelines.

Consensus Guidelines for p-Tau Assessment at Autopsy	NIA-AA 2012 AD Consensus Guidelines	NINDS/NIBIB 2016 Consensus Criteria for CTE
Lower threshold of p-tau for clinical correlation?	Yes	No
Upper limit for sampling?	Yes	No
Clinical context required?	Yes	No
Other disease processes exclusionary?	Yes	No
Diagnosis implies mechanism?	No	Yes

To permit AD as a clinicopathologic entity, the AD guidelines require an intermediate degree or more of both p-tau and Aβ as a lower threshold. Five samples for p-tau immunostaining is a suggested upper limit for tissue sampling, with some articles suggesting that fewer samples may be acceptable. The AD guidelines specify an extent of “neuropathologic change” rather than a clinical diagnosis. AD is only suggested if the decedent had dementia dura life (and abundant proteinopathy). The guidelines are not used to predict a clinical state. If another pathological entity (e.g., frontotemporal lobar degeneration, prion disease) is present, AD is excluded. The AD guidelines specifically state that the disease mechanism is unknown, despite the many advances in AD research. In contrast, CTE criteria have no lower threshold. A single microscopic lesion is sufficient for diagnosis. No upper limit of sampling is specified, with sampling and immunostaining beyond standard methods being necessary in about 20% of cases. There is no requirement for dementia or other clinical problems when applying the NINDS/NIBIB criteria. Any clinical context is acceptable. No disease process, neurodegenerative or otherwise, is exclusionary. The CTE diagnosis also implies a specific mechanism (neurotrauma), and is a diagnosis beyond doubt, i.e., pathognomonic, per the consensus criteria.
